# Assertive community treatment for high-utilizing alcohol misuse patients: a before-and-after cohort study protocol

**DOI:** 10.1186/s12913-023-10516-5

**Published:** 2024-02-28

**Authors:** Juntian Wu, Fahad Javaid Siddiqui, Charles Chia Meng Mak, Ivan Si Yong Chua, Jeevan Raaj Thangayah, Esther Xi Xiang Tan, Huey Ying Seet, Adriel Kailing Rao, Hann Yee Tan, Asif Mohamed, Mikael Hartman, Benjamin Sieu-Hon Leong, Marcus Eng Hock Ong, Desmond Renhao Mao

**Affiliations:** 1 Health Services Research Centre, SingHealth, Outram Singapore; 2 Health Services and Systems Research, Duke-NUS Medical School, Outram, Singapore; 3 Yong Loo Lin School of Medicine, National University of Singapore, Kent Ridge, Singapore; 4 Pre-hospital and Emergency Research Centre, Duke-NUS Medical School, Outram, Singapore; 5 National Addictions Management Service, Institute of Mental Health, Buangkok, Singapore; 6 Department of Emergency Medicine, Singapore General Hospital, Outram, Singapore; 7 Department of Emergency Medicine, Ng Teng Fong General Hospital, Jurong East, Singapore, Singapore; 8 Acute and Emergency Care Centre, Khoo Teck Puat Hospital, Yishun, Singapore; 9 Department of Geriatric Medicine, Tan Tock Seng Hospital, Novena, Singapore; 10 Department of Surgery, National University Hospital, Kent Ridge, Singapore; 11 Saw Swee Hock School of Public Health, National University of Singapore, Kent Ridge, Singapore; 12 Department of Emergency Medicine, National University Hospital, Kent Ridge, Singapore; 13 Unit for Pre-hospital Emergency Care, Ministry of Health, Outram, Singapore

**Keywords:** Alcohol-related frequent attenders, Emergency department utilization, Assertive community treatment, Healthcare resource optimization, Singapore

## Abstract

**Background:**

The challenge posed by Alcohol-Related Frequent Attenders (ARFAs) in Emergency Departments (EDs) is growing in Singapore, marked by limited engagement with conventional addiction treatment pathways. Recognizing this gap, this study aims to explore the potential benefits of Assertive Community Treatment (ACT) - an innovative, community-centered, harm-reduction strategy—in mitigating the frequency of ED visits, curbing Emergency Medical Services (EMS) calls, and uplifting health outcomes across a quartet of Singaporean healthcare institutions.

**Methods:**

Employing a prospective before-and-after cohort design, this investigation targeted ARFAs aged 21 years and above, fluent in English or Mandarin. Eligibility was determined by a history of at least five ED visits in the preceding year, with no fewer than two due to alcohol-related issues. The study contrasted health outcomes of patients integrated into the ACT care model versus their experiences under the exclusive provision of standard emergency care across Hospitals A, B, C and D. Following participants for half a year post-initial assessment, the evaluation metrics encompassed socio-demographic factors, ED, and EMS engagement frequencies, along with validated health assessment tools, namely Christo Inventory for Substance-misuse Services (CISS) scores, University of California, Los Angeles (UCLA) Loneliness scores, and Centre for Epidemiologic Studies Depression Scale Revised (CESD-R-10) scores.

**Discussion:**

Confronted with intricate socio-economic and medical challenges, the ARFA cohort often grapples with heightened vulnerabilities in relation to alcohol misuse. Pioneering the exploration of ACT’s efficacy with ARFAs in a Singaporean context, our research is anchored in a patient-centered approach, designed to comprehensively address these multifaceted clinical profiles. While challenges, like potential high attrition rates and sporadic data collection, are anticipated, the model’s prospective contribution towards enhancing patient well-being and driving healthcare efficiencies in Singapore is substantial. Our findings have the potential to reshape healthcare strategies and policy recommendations.

**Trial Registration:**

ClinicalTrials.gov, NCT 04447079. Initiated on 25 June 2020.

**Supplementary Information:**

The online version contains supplementary material available at 10.1186/s12913-023-10516-5.

## Background

Globally, in 2016, alcohol consumption ranked as the seventh primary cause for both mortality and Disability-Adjusted Life Years (DALYs), attributing to 2.2% of deaths in women and 6.8% in men. Almost one-tenth of worldwide deaths among the age group 15–49 years can be ascribed to alcohol consumption [1].

Patients who frequently utilize emergency department (ED) services due to alcohol-related issues are referred to as alcohol-related frequent attenders (ARFAs) [2]. These patients, often characterized by chronic alcohol misuse, constitute a small fraction of the ED population but disproportionately consume substantial emergency medical resources, including emergency medical services (EMS), EDs, and inpatient services [3, 4].

ARFAs not only present with acute symptoms of alcohol intoxication but also the long-term effects of alcohol misuse such as withdrawal symptoms, gastrointestinal bleeding, and complications of liver cirrhosis. Given the time-sensitive nature of EDs and the competing priorities therein, physicians often struggle to provide sustainable and long-term interventions to curb alcohol misuse in these patients [5].

Despite recommended treatment regimens for addiction, ARFAs frequently default, particularly within the initial three months. Data has suggested that this group of patients have high drop-out rates when referred for treatment [6]. Instead, this patient group often reverts to utilizing the ED and EMS, resulting in unplanned hospital admissions due to alcohol-related complications.

The acute and chronic presentations of ARFAs pose significant challenges, requiring substantial resources for close monitoring to prevent falls, dealing with potential staff abuse, cleaning services, and handling prolonged stays in crowded departments [7, 8]. Moreover, chronic alcohol misuse often leads to high-risk conditions like variceal bleeding and liver failure, thus escalating morbidity and mortality rates [9].

While extensive research on interventions for ARFAs [10], predominantly Case Management (CM), has been conducted in Western countries [11–13], few have explored the impact of assertive community treatment (ACT): a patient-focused, community-based approach which emphasizes the delivery of comprehensive and personalized services to patients.Despite a rigorous evidence base for CM, it has demonstrated variable reductions in ED use by ARFAs [14]. 

There is also a paucity of understanding regarding the characteristics of ARFAs and their response to ACT intervention.

This paper describes the study protocol of an interventional trial designed to investigate the effectiveness of ACT in managing ARFAs. We aim to detail the methods and considerations guiding the investigation, particularly focusing on the utilization of ACT within local contexts. The protocol considers the multifaceted needs of ARFAs, proposing comprehensive, community-based interventions that extend beyond traditional ED care.

Our intention is not only to present a structured and replicable study design but also to contribute to the ongoing discourse surrounding alcohol misuse treatment. By outlining the protocol in this paper, we seek to facilitate a broader understanding of ACT’s potential impact on ARFAs, offering insights that may inform future initiatives, policies, and research endeavours in managing problematic drinking behaviours, thus reducing dependency on EDs.

## Methods

### Study design

**Fig. 1:**
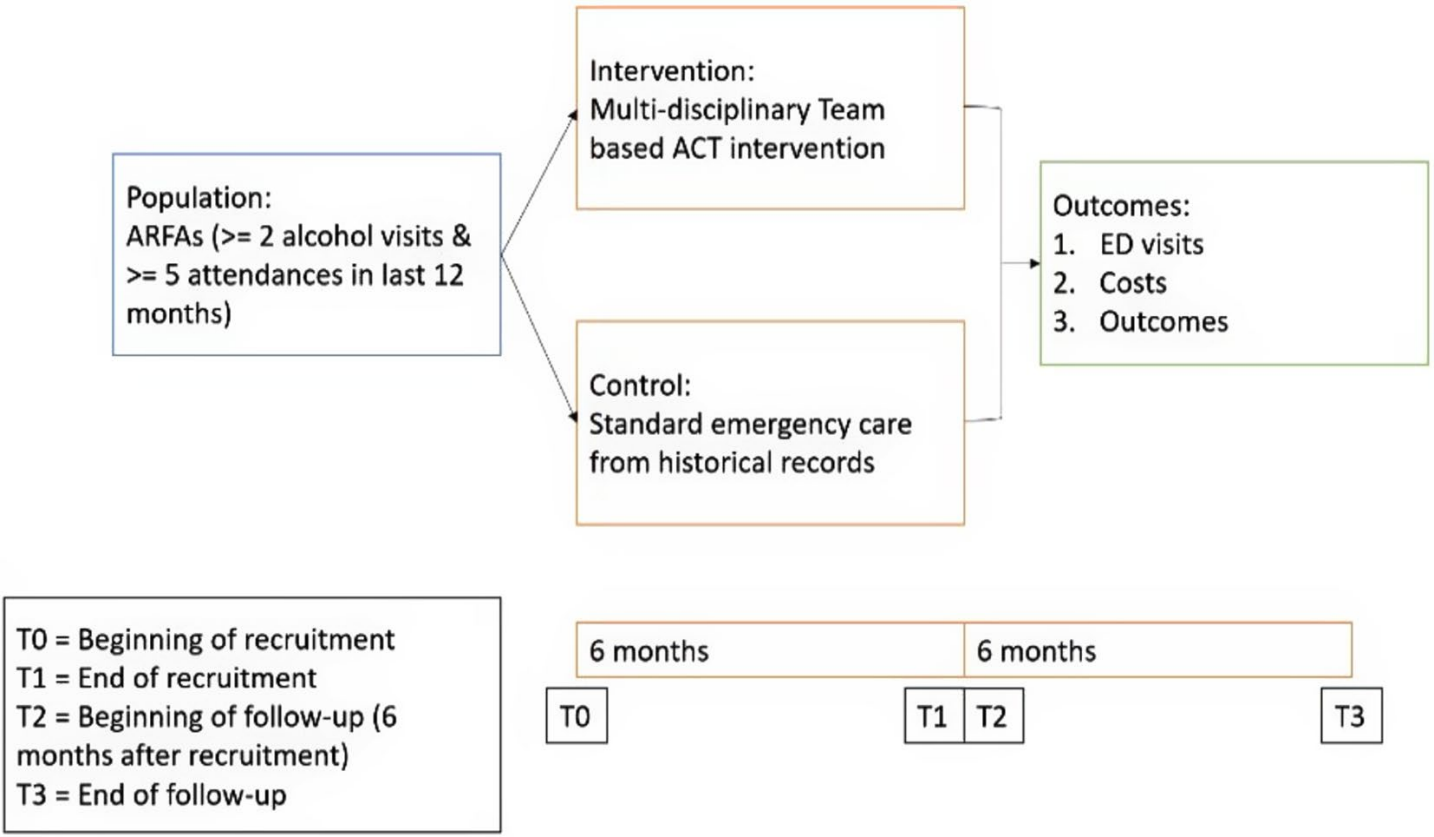
Study design with inclusion and follow-up timetable

This study is conducted as a prospective, multi-centre, before-and-after cohort study, comparing the outcomes of ACT interventions to standard emergency care among ARFAs from four adult EDs across Singapore (see Fig. 1).

### Objectives

1. To conduct a before-and-after trial of ARFAs in four public hospitals under three healthcare clusters in Singapore.
2. To evaluate the effectiveness and cost-effectiveness of ACT intervention for ARFAs as compared to standard care in the emergency departments.
3. To describe the patient characteristics, ED care characteristics and health outcomes of ARFAs.

### Primary hypothesis

1. ACT is expected to be more effective than standard care in reducing the annual number of ED visits by ARFAs, as measured at a 12-month follow-up.

### Secondary hypotheses

1. ACT is expected to improve health outcomes compared with standard care at a 12-month follow-up.
2. ACT is expected to be more cost-effective compared with standard care at a 12-month follow-up.

The study protocol comprises a total of 17 visits, including a follow-up at six months post-final assessment. The intervention manual and comprehensive study protocol is adhered to the Standard Protocol Items: Recommendations for Interventional Trials (SPIRIT) attached in Additional file 1 (see Additional file 1) and can be made available upon request from the authors.

Employing a before-and-after study design will facilitate measurements of ACT outcomes in ARFAs before and after its formal implementation in EDs. Changes in ARFAs’ outcomes will thereby be attributed to the ACT intervention. Previous ARFAs ED usage data serves as a control group for comparative analysis. While this design does not negate the need for randomization, it offers a more pragmatic approach that can be less resource-demanding than a randomized-controlled trial (RCT). Importantly, the study can be executed concurrently with standard ED operations.

### Participants

#### Inclusion criteria

Patients at the study site meeting the following criteria are eligible for participation: aged 21 years or older, have recorded a minimum of two alcohol-related and five all-cause visits to an ED in the preceding 12 months, and are fluent in either English or Mandarin. Patients who discontinued ACT but have completed a minimum of three ACT visits are included, considering that significant interventions would have been implemented into their social, drinking, and medical conditions by the third visit.

#### Exclusion criteria

Patients are excluded from the study if they are unable to provide informed consent or are ineligible to available ACT services. Furthermore, those who are not proficient in English or Mandarin, or are under the age of 21 years are excluded. Patients already engaged with ACT-related interventions or alcohol addiction programs from any other institution within Singapore are also excluded.

#### Screening and recruitment

Patients who meet the inclusion criteria are identified through clinical referrals within each participating hospital using alcoholism codes ICD-10-AM, SNOMED and ICD-9-CM codes. Each hospital’s ACT teams prioritize recruitment for those who have most frequently used ED services, with the aim to recruit up to 17 patients over six months (equating to 34 patients per year).

Four hospitals participate in the study over a four-year period (see Table 1). Recruitment of participants for each site is done yearly and staggered. Considering Singapore’s limited resources, and the existence of only one tertiary addiction centre, the study initiation dates are strategically staggered to ensure sufficient supervisory support.

**Table 1:** Details of recruitment

Intervention Site	Date of Initiation	Date of completion	Maximum Subjects
Hospital A	August 2020	February 2024	136
Hospital B	August 2021	February 2024	102
Hospital C	August 2022	February 2024	68
Hospital D	August 2023	February 2024	34

Invitations to partake in the study are extended via phone calls, offering potential subjects a week for consideration. However, pilot data suggest a significant proportion of ARFAs are homeless and lack phone access. As such, an opportunistic recruitment approach is primarily adopted, enabling engagement with eligible patients during their hospital visits. Members of the study team approach eligible patients, extending an invitation to participate and providing necessary information, including the need for retrospective data sharing consent. The consent is obtained by either Site-Principal Investigators (PIs) or Co-Investigators (Co-Is).

To optimize participant retention and ensure complete follow-up, we have implemented strategies that emphasize flexibility and participant engagement. Regular check-ins, either by phone or text, will maintain consistent communication, while appointment schedules are tailored to participants’ availabilities. If participants discontinue or deviate from the intervention protocol, we will attempt an exit interview to gather insights and continue collecting relevant outcome data, ensuring that their contribution remains valuable and meaningful to the study.

#### Trial flow and sample size

**Fig. 2:**
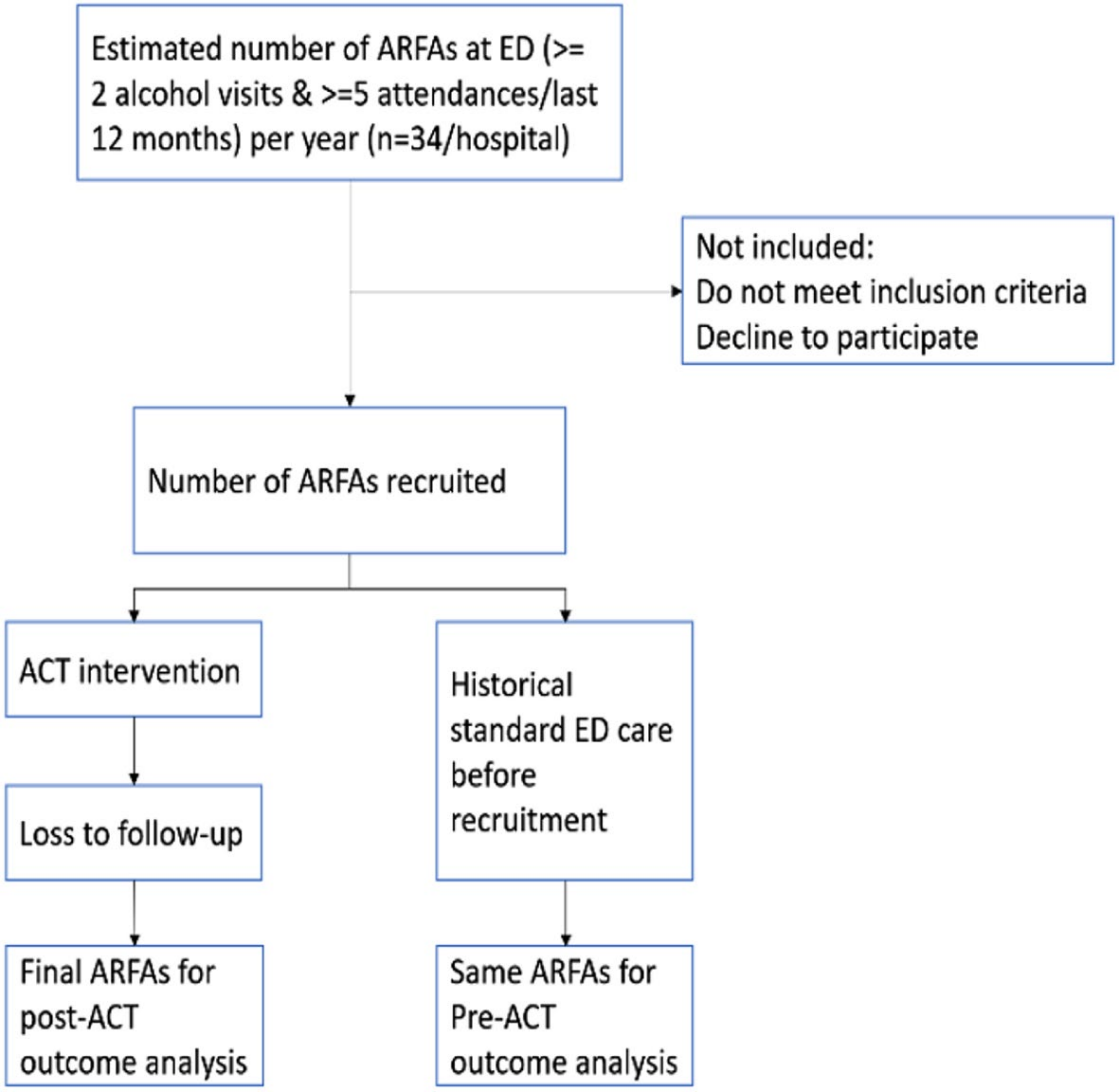
Study flow diagram

A detailed flow diagram is provided (see Fig. 2), demonstrating the progression through the multidisciplinary team-based ACT interventions, occurring parallelly to standard emergency care for ARFAs. The diagram’s numerical annotations are informed by our power analysis during the study design phase. As a real-world implementation study, our recruitment goal encompasses all patients in the ARFA program. According to past data, ARFAs utilize ED services at a mean of 30.15 visits/year, with a standard deviation (SD) of 23.47. With an expected 40% usage decrease, a standard deviation of 23 visits, 80% power, and an alpha of 0.05, we estimate a required sample size of 29 patients. Anticipating a 15% attrition rate, our target recruitment number stands at 34 patients.

### Intervention methods

#### ACT intervention

ACT will incorporate the following elements [15]:

i. Assertive engagement: assertively establishing contact and seeing patients in their homes and/or community settings.
ii. Holistic care: addressing physical/mental health and social care needs in a patient-led manner.
iii. Building relationships: establishing and maintaining contact, building rapport, working with, and supporting family members.
iv. Flexibility: working flexibly with patients’ goals even when their problems seem to be peripheral to addition.
v. Openness: being explicit about goals in care planning.
vi. Going out of your way: following an ethos of stepping outside of professional roles and going the extra mile for patients.
vii. Close follow-up: low staff-to-patient ratio to allow for effective case management and short intervals between reviews.
viii. Extended care: intensive case management for 6 months, establishing links with community services and resources, to facilitate long term support.

ACT will be used to proactively engage with ARFA patients, to assess their physical and mental health and wider social needs, then to develop shared treatment goals, and to facilitate and coordinate engagement with the relevant care and support to address their multiple presenting needs. The ACT will mainly take place either in the patient’s home or local community settings.

#### The multidisciplinary team-based ACT intervention

Historical patient data are collected from the electronic health record system. We implement a multidisciplinary, team-based ACT intervention, targeting medical, psychiatric/psychological, social and substance (MPSS) domains. The ACT teams follow a predefined schedule, with each engagement spanning approximately 60 min. Interactions include CM and collaboration with a social service agency (SSA). Patients who opt out of the intervention will still be considered in the analysis.

The ACT team will maintain active engagement with ARFAs on a schedule detailed (see S Table 1), with a typical interaction lasting 60 min.

Each visit is not rescheduled in the event of a patient’s absence; instead, the team will re-engage the patient via phone to plan for the succeeding visit as per schedule. Consistent with the core principles of ACT, ARFAs are assertively engaged in the community, receiving intensive, comprehensive, and supportive care with a harm reduction approach. This also incorporates CM and collaboration with a SSA.

**Fig. 3:**
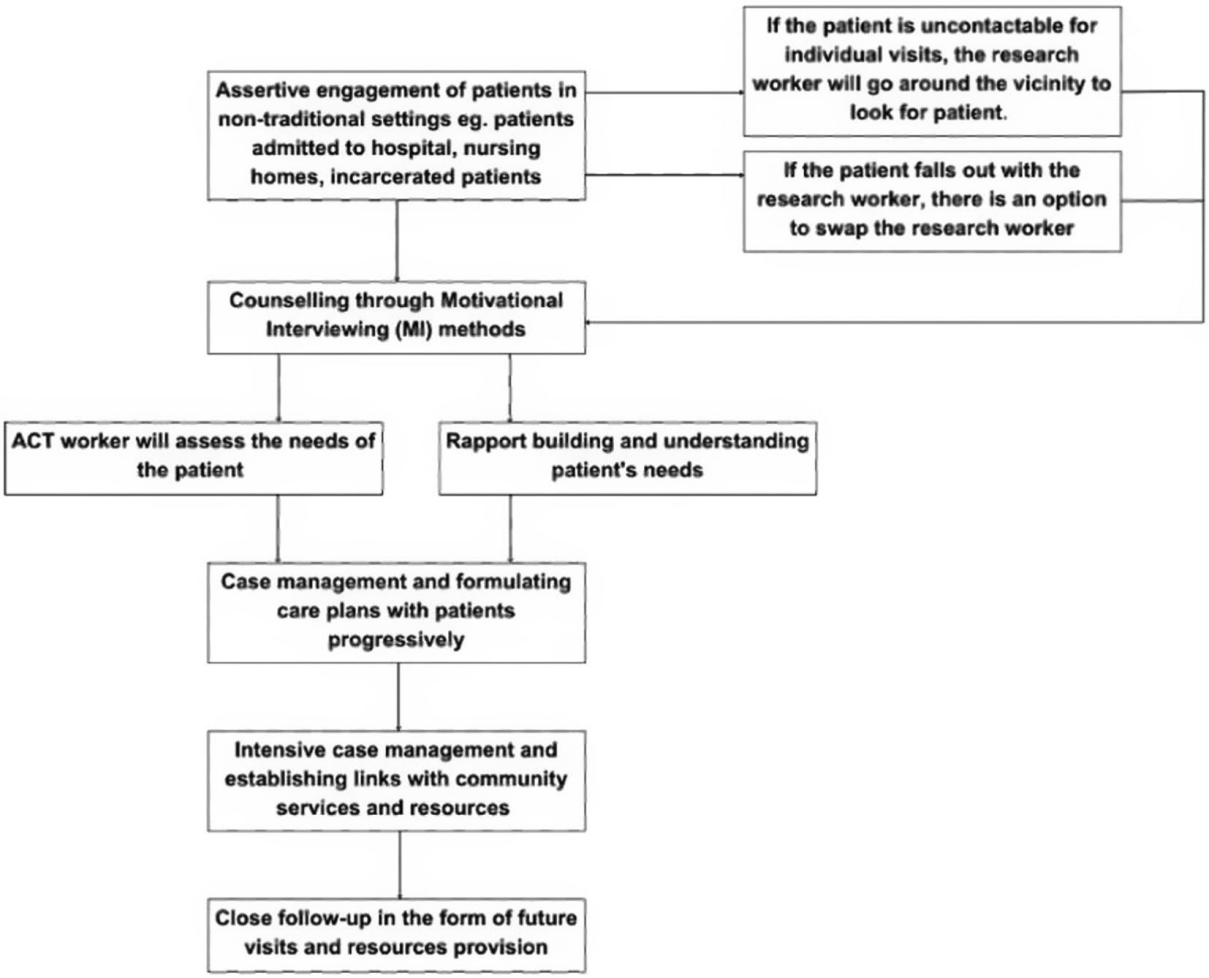
Example of a typical ACT visit

A typical ACT visit is as outlined in Fig. 3. “Assertive” in ACT means ACT workers lean forward in terms of patient recruitment and retention. This involves visiting patients in various non-traditional settings including but not limited to hospital admissions, nursing homes and incarceration. If the patient is uncontactable for individual visits, the research worker will go around the vicinity to look for the patient. In the event that a patient falls out with the research worker, an option to switch the research worker is available, as it is a counselling-based intervention.

Each ACT intervention interaction encompasses various facets of physical health, mental health, and addiction. The ACT team will address problems across the four life domain categories of MPSS. Weekly multi-disciplinary team (MDT) meetings will be held for problem discussions following the MPSS format.

Given the community-based nature of ACT, ARFAs may be encountered at various locations beyond their residences. Privacy is prioritized during all interactions. ARFAs are briefed about the limitations of confidentiality, and consent is obtained prior to each session. CM elements, including phone call reminders for medical appointments, psychoeducation, and medication assistance, will be provided.

#### Teamwork, training, and continuing education

The core ACT team, consisting of physicians, nurses, and allied health practitioners, receive training and support from national addictions management service.

The team members undergo intensive training in motivational interviewing and cross-cultural competencies, along with specific courses on adequate social assistance referral. Formal training sessions are conducted prior to the launch of ACT services at each participating hospital, with a stipulated data collection schedule attached in Additional file 2 (see Additional file 2).

#### Data monitoring

Our study is overseen by ARFA steering committee consisting of PIs and Co-PIs of this study. The committee ensures the safety and efficacy of data collection and the ethical conduct of the trial. No members of the committee have competing interests in the trial’s outcomes. Interim analyses are conducted during ARFA’s follow-up. The primary purpose of these analyses is to assess safety parameters and to check for any unforeseen risks. An independent audit will be conducted yearly by the hospitals to ensure the trial’s conduct aligns with the approved protocol and good clinical practice guidelines.

#### Primary outcome: number of ED visits

The primary outcome is the number of ED visits by ARFAs, sourced primarily from the electronic records system of the participating hospitals. A comparative analysis will be conducted between the rates of ED visits pre-intervention (12 months) and post-intervention (12 months).

#### Secondary outcomes: cost analysis & health outcomes

1. *Cost Analysis*: The secondary outcome will first focus on the cost implications of healthcare resource consumption by ARFAs. We will analyse the costs generated at participating hospitals but cannot incorporate costs incurred at non-participating community institutions. We will calculate the net cost savings by comparing the cost of ACT during the intervention period against the cost savings from decreased ED encounters in the 12 months post-intervention.
2. *Health Outcomes*: We will measure ARFA outcomes using the Christo Inventory of Substance-misuse Services (CISS) score, a validated marker of alcohol dependence severity, and the UCLA 3-point Loneliness Scale, a quick method for assessing social isolation. Furthermore, we will use the Center for Epidemiologic Studies Depression Scale Revised (CESD-R-10) to measure depression. Comparisons will be drawn between pre-ACT and post-ACT scores after six months of treatment.

### Statistical methods

Continuous variables will be reported as mean and standard deviation using Student’s t-test. Categorical variables will be reported as frequency count and percentage using Chi-square test/Fisher’s exact test. Univariate analysis will be done to select variables for an adjusted multivariable analysis. Statistical significance will be set at P < 0.05 criteria. We will adopt imputation to address missing data.

Using complete set data analysis, cases undergoing ACT will be compared their own historical outcomes as controls. ED utilizations will be compared using the paired sample t-test or Wilcoxon Signed Rank test, the post-intervention utilization of ARFAs undergoing ACT will be compared to the pre-intervention utilization.

To adjust for potential confounders, multivariable regression analysis will be undertaken. Interactions will be tested for variables that are clinically relevant and are statistically significant. Model adequacy will be assessed through coefficients of determination (R2) and by plotting error terms against predicted values. Presence of multicollinearity will be evaluated by variance inflation factor (VIF). Should complete set analysis show efficacy, we will proceed to analyze the data using an intention-to-treat analysis.

The incremental costs of delivering the intervention will be estimated and compared to any observed cost savings from reduce usage of ED services and other health services. All analyses will be performed using R 4.2.0 (www.r-project.org) and STATA version 17.0 (StataCorp. 2017. Stata Statistical Software: Release 17.0. College Station, TX: StataCorp LLC) software.

### Preliminary results

A total of 93 ARFAs have been recruited from three participating hospitals as of March 2023 (see S Table 2): Hospital A (n = 37), Hospital B (n = 41), and Hospital C (n = 15). A broad age range was represented in the study with a median of 57 years and an Interquartile Range (IQR) of 51–64 years across all hospitals. It is noteworthy that Hospital C reported the highest median age of 61. In terms of age distribution, the majority of patients (76.34%) were below 65 years of age, and Hospital A reported the greatest proportion (86.49%) in this category.

The study cohort was predominantly male, accounting for 93.55% of the overall sample. Ethnic diversity was observed among the patients; however, Indian ethnicity was the most prevalent, making up 63.44% of the total sample.

In the context of marital status, a substantial proportion of patients were divorced (36.56%), with Hospital A having the highest prevalence of divorce (48.65%). On the contrary, Hospital C had a distinctively higher percentage of single patients (53.33%) compared to other hospitals.

The living arrangements of patients varied significantly across the three hospitals. Overall, 41.94% of patients lived in their own or a family-owned home. An outlier was observed in Hospital C, where the majority of patients (73.33%) lived in a rental flat, a considerably higher percentage than that reported by the other hospitals.

Assessing employment status revealed that a significant majority of the cohort was unemployed (66.67%), a trend consistently seen across all hospitals. Furthermore, smoking was common among the patient population, with an overall prevalence of 81.72%.

When examining medical histories, liver disease emerged as the most frequently reported condition, affecting more than half (52.69%) of the patients. A noteworthy observation was the significantly higher prevalence of substance abuse in Hospital C (60%), a finding that was statistically significant across the hospitals (p = 0.004).

## Discussion

Our study is poised to offer an unprecedented exploration of an ACT-based approach applied to ARFAs in Singapore. This initiative is unique in its distinctive focus on a complex patient demographic, characterised by pervasive alcoholism intricately enmeshed with psychosocial challenges. Conventionally, this subgroup is typically overlooked within the realm of ED, therefore, our research is set to offer substantial insights into the broader implications of ARFAs on Singapore’s health system.

The cornerstone of our study’s uniqueness is the explicit focus on the recruitment of high-utilizing patients within the healthcare system. These patients, often resistant to traditional forms of treatment due to a range of obstacles, such as homelessness, lack of constant means of communication, and a tendency towards disengagement, represent a significant challenge to healthcare providers. The strategic navigation of these barriers through an innovative and assertive recruitment approach sets our study apart in the realm of addiction studies.

A further distinctive aspect of our study is the implementation of the ACT intervention. A significant departure from conventional treatment methods, ACT adopts a patient-centric strategy that assertively engages ARFAs within their own community settings. The intervention’s multifaceted approach acknowledges the importance of a holistic recovery pathway that addresses the diverse issues facing ARFAs, inclusive of medical, psychological, social, and alcohol-related domains. The potential for novel outcomes presented by the ACT intervention’s focus on case management presents a fresh lens through which patient recovery can be perceived.

Regarding methodology, our study employs a before-and-after cohort study design. This strategic approach enables a comprehensive comparison of patient outcomes pre and post-intervention, effectively measuring the impact of the ACT intervention on a broad array of factors, from emergency healthcare utilization to patient psychosocial wellbeing and overall health status. We employ recognised and validated tools such as the CISS, UCLA Loneliness Scale, and the CESD-R-10, enhancing the rigour, reliability, and generalisability of our study’s outcomes.

An examination of the demographic data of the study population underscores the diverse nature of the ARFAs. The participants span a broad age spectrum, with a significant majority being males. Importantly, the ethnic diversity in the patient cohort, particularly the high prevalence of Indian ethnicity, mirrors the multicultural context of Singapore and potentially the complex socio-cultural factors at play in ARFA. The marital and living arrangement statuses underscore the complex social realities of this patient population. A significant proportion of ARFAs were divorced and living in rental flats or were homeless, indicative of the potential socioeconomic adversities they face. The remarkably high unemployment rate among the ARFAs further accentuates these hardships.

From the health perspective, the high prevalence of smoking, liver disease, and other medical conditions like pancreatitis, seizures/epilepsy, gastritis, and notably substance abuse, further delineate the complex clinical profile of ARFAs. These comorbidities could potentially amplify the difficulties in managing alcoholism, necessitating a comprehensive and integrated care approach like ACT. The ACT intervention, a novel approach in Singapore’s context, promises to provide holistic care to these patients in community-based settings. By addressing their medical, psychological, social, and alcohol-related problems, ACT aims to assist these patients in achieving overall well-being. This study could illuminate the potential benefits and challenges associated with implementing such an approach.

In spite of the significant advantages our study brings to the field, we also acknowledge potential limitations and challenges. One of the anticipated barriers is the high dropout rate due to the recurrent nature of addiction. To mitigate this issue, we intend to maintain consistent follow-ups with this typically disengaged patient group and establish robust tracking and engagement strategies tailored to this unique population.

A further complication arises from ACT itself, which may result in decreased visits and an inability to complete the intervention. A patient appropriately institutionalized through ACT may lead to a loss of contact due to lack of access. To overcome this obstacle, we plan to foster collaboration with participating institutions and community partners, educating them about our research and establishing protocols for continued patient engagement. This coordinated approach aims to ensure that access to participants is maintained even if they are transferred to institutional care.

Difficulty in data collection is another expected barrier. Patients’ decreased brain function, inability to concentrate, and possible mild intoxication may result in missing data. To address this challenge, we will implement a flexible data collection approach, allowing for information to be gathered during subsequent visits if initial attempts are unsuccessful. Furthermore, we will employ trained researchers adept at working with this specific population, thereby maximizing the chances of obtaining complete and accurate data.

Notwithstanding these challenges, the potential for the ACT model to significantly enhance ARFAs’ physical and psychosocial well-being cannot be understated. The possibility of cost-savings and improved resource allocation within the healthcare system presents a compelling argument for further exploration of this model. Our study, guided by a pragmatic design, and informed by clear strategies to navigate these challenges, aims to contribute valuable insights into the effective treatment and management of ARFAs. We believe that our thoughtful approach to these barriers, combined with the innovative nature of our study, will ultimately allow us to successfully complete this important research.

The findings from our study are expected to be instrumental in informing healthcare frameworks and policy-making. Our research will provide critical evidence on cost-effectiveness, improved resource utilization, and potential for overall cost savings for Singapore’s healthcare system. With rising healthcare costs and demands, these insights could be transformational in enhancing the efficiency of emergency healthcare services.

In conclusion, our study, with its unique patient focus, innovative intervention strategy, and robust analysis techniques, represents a significant contribution to the field of addiction studies. As we seek to navigate the complex landscape of managing ARFAs, this research offers a novel perspective with the potential to redefine best practices, inform healthcare policy, and ultimately improve patient outcomes.

### Electronic supplementary material

Below is the link to the electronic supplementary material.

**Supplementary Material 1:** SPIRIT Checklist

**Supplementary Material 2:** Data Collection Schedule

**Supplementary Material 3:** Schedule of ACT engagement

**Supplementary Material 4:** Preliminary Results of Characteristics of Alcohol-Related Frequent Attenders at 3 Institutions

## Data Availability

The datasets generated and/or analysed during the current study are not publicly available due to confidentiality of the collected data but are available from the corresponding author on reasonable request.

## Abbreviations

ARFA: Alcohol Related Frequent Attenders
ACT: Assertive Community Treatment
ED: Emergency Department
EMS: Emergency Medical Service
CISS: Christo Inventory for Substance-misuse Services
UCLA: University of California, Los Angeles
CESD-R-10: Centre for Epidemiologic Studies Depression Scale Revised
DALY: Disability-Adjusted Life Years
CM: Case Management
ICD-10-AM: International Classification of Diseases and Related Health Problems, Tenth Revision, Australian Modification
SNOMED: Systemised Nomenclature of Medicine
ICD-9-CM: International Classification of Diseases, Ninth Revision, Clinical Modification
MPSS: Medical, Psychiatric/Psychological, Social, and Substance
SSA: Social Service Agency
MDT: Multi-Disciplinary Team
