# Supplementary material for: Assertive community treatment for high-utilizing alcohol misuse patients: a before-and-after cohort study protocol
Source: BMC Health Serv Res. 2024 Feb 28;24:256. doi: 10.1186/s12913-023-10516-5 (PMC10900701; doi:10.1186/s12913-023-10516-5)
Supplement: Supplementary file 2 — Supplementary Material 2: Data Collection Schedule [file 12913_2023_10516_MOESM2_ESM.docx]

**Additional file 2: Data Collection Schedule**

The data collection schedule from the point of recruitment, eligibility screen, enrolment, consent to the intervention and follow-up visits are outlined below. Specific data related to the patient’s CISS, UCLA ad CESD-R scores, which are required for collection are also outlined at different time points as shown in the table below.

**Table 1.** Data collection schedule

| **STUDY PERIOD** | | | | | | | | | | | | | | | | | | | |
| --- | --- | --- | --- | --- | --- | --- | --- | --- | --- | --- | --- | --- | --- | --- | --- | --- | --- | --- | --- |
|  | **Recruitment** | **Allocation** | **Post-allocation** | | | | | | | | | | | | | | | | **Close-out** |
| **TIMEPOINT**** | ***-t_1_*** | ***t_0_*** | ***t_1_*** | ***t_2_*** | ***t_3_*** | ***t_4_*** | ***t_5_*** | ***t_6_*** | ***t_7_*** | ***t_8_*** | ***t_9_*** | ***t_10_*** | ***t_11_*** | ***t_12_*** | ***t_13_*** | ***t_14_*** | ***t_15_*** | ***t_16_*** | ***t_17_*** |
| **ENROLMENT:** |  |  |  |  |  |  |  |  |  |  |  |  |  |  |  |  |  |  |  |
| **Eligibility screen** | X |  |  |  |  |  |  |  |  |  |  |  |  |  |  |  |  |  |  |
| **Invitation to Participate** | X |  |  |  |  |  |  |  |  |  |  |  |  |  |  |  |  |  |  |
| ***Informed Consent*** |  | X |  |  |  |  |  |  |  |  |  |  |  |  |  |  |  |  |  |
| **INTERVENTION:** |  |  |  |  |  |  |  |  |  |  |  |  |  |  |  |  |  |  |  |
| ***ACT*** |  |  |  |  |  |  |  |  |  |  |  |  |  |  |  |  |  |  |  |
| **ASSESSMENTS:** |  |  |  |  |  |  |  |  |  |  |  |  |  |  |  |  |  |  |  |
| ***Cover Page*** |  |  | X |  |  |  |  |  |  |  |  |  |  |  |  |  |  |  |  |
| ***Baseline Visit*** |  |  | X |  |  |  |  |  |  |  |  |  |  |  |  |  |  |  |  |
| ***Follow-up Visit*** |  |  |  | X | X | X | X | X | X | X | X | X | X | X | X | X | X | X | X |
| ***CISS*** |  |  | X |  |  | X |  |  |  | X |  |  | X |  | X | X | X | X | X |
| ***UCLA*** |  |  | X |  |  | X |  |  |  |  |  |  | X |  |  |  |  | X |  |
| ***CESD-R*** |  |  |  | X |  |  |  | X |  |  |  | X |  |  | X |  |  |  |  |
| ***Final Visit*** |  |  |  |  |  |  |  |  |  |  |  |  |  |  |  |  |  |  | X |
