# Supplementary material for: Assertive community treatment for high-utilizing alcohol misuse patients: a before-and-after cohort study protocol
Source: BMC Health Serv Res. 2024 Feb 28;24:256. doi: 10.1186/s12913-023-10516-5 (PMC10900701; doi:10.1186/s12913-023-10516-5)
Supplement: Supplementary file 3 — Supplementary Material 3: Schedule of ACT engagement [file 12913_2023_10516_MOESM3_ESM.docx]

**S Table 1.** Schedule of ACT engagement

| **ACT Visit** | **Period of ACT Visit** | **Remarks** |
| --- | --- | --- |
| 1 | Week 0 |  |
| 2 | Week 1 | One week after Visit 1 |
| 3 | Week 2 |  |
| 4 | Week 3 |  |
| 5 | Week 4 |  |
| 6 | Week 5 |  |
| 7 | Week 6 |  |
| 8 | Week 7 |  |
| 9 | Week 8 |  |
| 10 | Week 10 | 2 weeks after Visit 9 |
| 11 | Week 12 |  |
| 12 | Week 15 | 3 weeks after Visit 11 |
| 13 | Week 18 | Combined visit with SSA  Interim assessment to decide ARFA’s disposition based on ED visits |
| 14 | Week 21 | Combined visit with SSA |
| 15 | Week 24 | Combined visit with SSA |
| 16 | Week 26 | Final visit from ACT team. To discharge to SSA or addiction services. |
| 17 | Week 52 | Follow-up visit from ACT Team |

SSA: Social Service Agency

S Table 1 should be placed in page 14, after lines 237-238 “The ACT team will maintain active engagement with ARFAs on a schedule detailed (see S Table 1), with a typical interaction lasting 60 minutes. “
