# Supplementary material for: Assertive community treatment for high-utilizing alcohol misuse patients: a before-and-after cohort study protocol
Source: BMC Health Serv Res. 2024 Feb 28;24:256. doi: 10.1186/s12913-023-10516-5 (PMC10900701; doi:10.1186/s12913-023-10516-5)
Supplement: Supplementary file 4 — Supplementary Material 4: Preliminary Results of Characteristics of Alcohol-Related Frequent Attenders at 3 Institutions [file 12913_2023_10516_MOESM4_ESM.docx]

**S Table 2.** Preliminary results of ccharacteristics of alcohol-related frequent attenders at 3 institutions

| **Parameter** | **Total**  **(N=93)** | **Hospital A**  **(n=37)** | **Hospital B**  **(n=41)** | **Hospital C**  **(n=15)** | **P** |
| --- | --- | --- | --- | --- | --- |
| **Age, Median (IQR)**  **Age category (%)**  Below 65  65 and above | 57 (51-64)  71 (76.34)  22 (23.66) | 55 (51-60)  32 (86.49)  5 (13.51) | 58 (53-67)  28 (68.29)  13 (31.71) | 61 (52-65)  11 (73.33)  4 (26.67) | 0.28  0.16 |
| **Gender (%)**  Male | 87 (93.55) | 34 (91.89) | 38 (92.68) | 15 (100) | 0.53 |
| **Ethnicity (%)**  Chinese  Malay  Indian  Eurasian  Others | 25 (26.88)  6 (6.45)  59 (63.44)  2 (2.15)  1 (1.08) | 9 (24.32)  2 (5.41)  24 (64.86)  1 (2.7)  1 (2.7) | 11 (26.83)  2 (4.88)  27 (65.85)  1 (2.44)  - | 5 (33.33)  2 (13.33)  8 (53.33)  -  - | 0.87 |
| **Marital status (%)**  Single  Married  Divorced  Widowed | 31 (33.33)  24 (25.81)  34 (36.56)  4 (4.3) | 4 (10.81)  13 (35.14)  18 (48.65)  2 (5.41) | 19 (46.34)  8 (19.51)  12 (29.27)  2 (4.88) | 8 (53.33)  3 (20)  4 (26.67)  - | 0.02 |
| **Living Arrangement (%)**  Own/Family-owned home  Rental flat  Homeless | 39 (41.94)  36 (38.71)  18 (19.35) | 16 (43.24)  12 (32.43)  9 (24.32) | 20 (48.78)  13 (31.71)  8 (19.51) | 3 (20)  11 (73.33)  1 (6.67) | 0.05 |
| **Employment Status (%)**  Employed  Unemployed  Retired | 20 (21.51)  62 (66.67)  11 (11.83) | 6 (16.22)  27 (72.97)  4 (10.81) | 10 (24.39)  24 (58.54)  7 (17.07) | 4 (26.67)  11 (73.33)  - | 0.37 |
| **Smoking Status (%)**  Yes | 76 (81.72) | 34 (91.89) | 27 (65.85) | 15 (100) | 0.002 |
| **Medical History (%)**  Liver disease  Pancreas disease  Seizure/Epilepsy  Gastritis  Substance abuse | 49 (52.69)  15 (16.13)  22 (23.66)  34 (36.56)  24 (25.81) | 22 (59.46)  7 (18.92)  10 (27.03)  17 (45.95)  6 (16.22) | 19 (46.34)  7 (17.07)  7 (17.07)  11 (26.83)  9 (21.95) | 8 (53.33)  1 (6.67)  5 (33.33)  6 (40)  9 (60) | 0.51  0.54  0.37  0.21  0.004 |

S Table 2 should be placed in page 19, after lines 330-335 “A total of 93 ARFAs have been recruited from three participating hospitals as of March 2023 (see Table 3): Hospital A (n=37), Hospital B (n=41), and Hospital C (n=15). A broad age range was represented in the study with a median of 57 years and an Interquartile Range (IQR) of 51-64 years across all hospitals. It is noteworthy that Hospital C reported the highest median age of 61. In terms of age distribution, the majority of patients (76.34%) were below 65 years of age, and Hospital A reported the greatest proportion (86.49%) in this category.”
